# Effect of Post-Cure on the Static and Viscoelastic Properties of a Polyester Resin

**DOI:** 10.3390/polym12091927

**Published:** 2020-08-26

**Authors:** Marco P. Silva, Paulo Santos, João M. Parente, Sara Valvez, Paulo N. B. Reis, Ana P. Piedade

**Affiliations:** 1 C-MAST, Department of Electromechanical Engineering, University of Beira Interior, Calçada Fonte do Lameiro, 6201-100 Covilha, Portugal; marco.silva@ubi.pt (M.P.S.); paulo.sergio.santos@ubi.pt (P.S.); joao.miguel.parente@ubi.pt (J.M.P.); sara.valvez@ubi.pt (S.V.); preis@ubi.pt (P.N.B.R.); 2 University of Coimbra, CEMMPRE, Department of Mechanical Engineering, 3030-788 Coimbra, Portugal

**Keywords:** polyester resin, post-cure, mechanical properties, mechanical testing

## Abstract

This work intends to study the effect of the curing parameters on the mechanical properties of a polyester resin without a complete curing reaction process. For this purpose, cures at room temperature, 40 °C, and 60 °C, and post-cures at 40 °C and 60 °C, with different exposure times, were considered. Three-point bending tests were performed to assess the bending properties and both stress relaxation and creep behavior. The degree of crosslinking was estimated by evaluating the C = C ester bond, by Fourier infrared spectroscopy and complemented with the thermal characterization made by differential scanning calorimetry. The results showed that higher curing temperatures are preferable to methods involving curing and post-curing, which can be confirmed by the higher degree of conversion of unsaturated ester bonds at 60 °C. Compared to the resin cured at room temperature, the bending strength increased by 36.5% at 40 °C and 88.6% at 60 °C. A similar effect was observed for bending stiffness. In terms of stress relaxation and creep strain, the lowest values were obtained for samples cured at 60 °C.

## 1. Introduction

Thermosetting resins are extensively used in diverse areas ranging from boats, consumer goods, aircraft, automotive components, and several industrial applications. Regarding the polyester resins, for example, they are the most widely used resins in polymer matrix composites, particularly in the maritime and automotive industries, and represent about 75% of total resin used in the composites industry. They are the most economical resin systems used in engineering applications that, simultaneously, combine low viscosity, adequate resistance to water and variety of chemicals, adequate resistance to weathering and ageing, good wetting to glass fibers, and relatively low shrinkage between 4 and 8% during curing. Nerveless, polyester resins have limited use in high-performance composites [1,2,3].

Regardless of their excellent aptitude for different manufacturing processes, such as compression molding, injection molding, resin transfer molding, and hand lay-up process [1,2], the main problem is related to their curing process [4,5,6]. The complexity of the problem is considerable, mainly in the quality control of the cure, because the process is affected by numerous factors, including ambient temperature, quality, and quantity of catalysts, the volume of the cured portion, and heat removal from the curing area. Therefore, both mechanical performance and surface appearance will be dramatically affected by the quality of the cure, a problem that is transversal to the various types of thermosetting resins [7,8].

The curing process of unsaturated polyester is exothermic due to a polymerization reaction that causes crosslinking among individual linear polymer chains [9,10,11,12]. Therefore, as polymerization by free radicals, the curing reaction of unsaturated polyester can be initiated by either the thermal or reduction–oxidation (redox) initiation system. Moreover, organic peroxides (initiators) can also initiate the reaction by adding metallic salts or amines (initiator + promoter system) at room temperature (RT). Unlike other thermosetting resins, no byproducts are formed during the curing reaction, and these resins can be molded, cast, and laminated at low pressures and temperatures [13,14,15,16]. However, many of the processing problems arise precisely in this curing process, which are generally ignored in the production planning phase. In this context, it is essential to have the curing data to correctly understand the final properties of polymers and/or composites and, thus, to ensure safe applications.

According to the open literature, different parameters define the post-cure process, but the post-cure temperature is considered the most important factor that influences the extent of crosslinking [8]. Senthilkumar et al. [17], for example, developed works about the temperature effect on the mechanical properties of sisal fiber reinforced polyester composites. For this purpose, composites were produced for different curing temperatures ranging from 60 to 100 °C in steps of 10 with a hot compression molding process. The best tensile and impact strength was obtained for composites cured at 100 °C. Kumar et al. [18] studied the post-curing temperature and time on the interlaminar shear strength (ILSS) and glass transition temperature (T_g_) of glass fiber reinforced polymer (GFRP) composites. Three different temperatures (80 °C, 110 °C, and 140 °C) and four different periods (2 h, 4 h, 6 h, 8 h, and 12 h) were analyzed. They concluded that post-curing at 140 °C for 6 h gave better thermal and mechanical properties as compared to post-curing at different temperatures and periods. For example, in terms of ILSS, a drastic increase was observed between 2 and 6 h, but, when the maximum was reached after 6 h, this property remains constant (a negligible effect was observed). According to the authors, the energy available at lower post-curing temperatures may not be enough to enhance further the wettability at the fiber/matrix interface.

In contrast, on the highest temperature, the energy received by the system is reasonably high for activating the monomers to crosslink further. Campana et al. [19] studied the effect of post-cure on flax fiber reinforced epoxy composites, and the following conditions were analyzed: composite cured at 80 °C for 24 h, 2 h at 100 °C, 120 °C, and 150 °C. They observed a crosslinking degree of 92% and a T_g_ of 121 °C for 24 h of curing at 80 °C. Nevertheless, when the resin was post-cured 2 h at 150 °C, these values increased to 100% and 165 °C, respectively. This trend was expected because the goal of post-cure is to achieve a crosslinking rate of 100%. After post-cure, the molecular mobility within the resin is reduced, leading to a higher T_g_. It was also noticed that the resin and composite followed the same trend, but, while the T_g_ of the composite was lower when cured at 80 °C for 24 h, all conditions that involved post-cured promoted higher values. This behavior is explained by a change in specific heat or thermal conductivity when the fibers were introduced [20,21]. Finally, Elleuch et al. [22] confirmed that a woven glass-polyester composite’s mechanical properties increased with the post-cure. The low crosslinking density effect increases the resin ductility and the failure strain. Therefore, a rigid interface (brittle failure) was obtained in the case of post-cure and a weak interface (progressive failure) in the case of post-cure absence.

Therefore, the main goal of the present work is to study the curing parameters of a polyester resin usually used on surf boards to understand their effects on mechanical properties. Moreover, due to some degree of ductility required for the specific application of this polyester resin, the curing and post-curing processes are recommended, by the manufacturer, to occur at much lower temperatures than the ones usually reported in the literature. Therefore, special attention will be given to the viscoelastic behavior of the resin because, due to its inherent viscoelasticity, composites will be prone to creep and stress relaxation, constituting a significant challenge when used in long-term applications.

## 2. Materials and Methods

**Figure 1:**
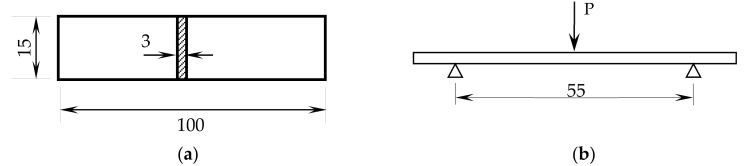
(**a**) Specimens geometry (dimensions in mm) and (**b**) schematic view of the three-point bending apparatus. All dimensions in mm.

A polyester resin (AROPOL FS 1962, SF Composites, Mauguio, France) was mixed with 1% peroxide (MEKP-50, SF Composites, Mauguio, France), and, after mixing, the system was degassed in a vacuum oven to remove any potential air bubbles. According to the manufacture, the resin presents low reactivity, which implies that after curing is almost inert towards other chemical species, and it is formulated with additives: a preaccelerating compound (enabling faster polymerization), and an UV stabilizer. The main properties/characteristics are (at RT): a viscosity of 550 mPa.s, a styrene content of 34%, and a geltime with 1.5% of MEKP-50 of 12 minutes. The crosslinking agent (or hardener) is a methyl ethyl ketone peroxide (MEKP-50) 33% (*v*/*v*) in dimethyl phthalate with a viscosity of 20 mPa.s, a peroxide content of 33%, and active oxygen content between 8.8 and 9%. After being degassed, the mixture was poured into a rectangular cardboard mold, with dimensions of 100 × 100 × 3 mm^3^, and between acetate sheets to both facilitate its removal after curing and obtaining an excellent surface finish. After unmolding the plates, they were submitted to different post-cures that are summarized in Table 1. Different temperatures were considered based on the supplier’s datasheet and the post-cure time based on the different values suggested in the literature [18,19]. Finally, the specimens were cut from those plates, with the final dimensions of 100 × 10 × 3 mm^3^, using a diamond saw and a moving speed chosen to reduce the heat in the specimen (Figure 1).

The analysis of the chemical functional groups of the resin, crosslinker, and cured samples was performed using Fourier transform infrared spectroscopy (FTIR) in the reflectance mode, using the non-destructive attenuated total reflectance (ATR) sampling technique.

The infrared spectrum (4000–550 cm^−1^) of the samples at room temperature was recorded using a Perkin Elmer Frontier spectrometer (FT NIR/MIR, PerkinElmer, Waltham, MA, USA), equipped with an FR-DTGS detector and a KBr beam splitter. Spectrum registration was performed with a 4.0 cm^−1^ resolution with 16 accumulations. A Perkin Elmer sampling accessory, universal ATR module (UATR universal attenuated total reflectance, PerkinElmer, Waltham, MA, USA) with diamond crystal/ZnSe, was used, and a constant 80 N force was applied throughout the spectrum register. The specimens were analyzed without any treatment and three repetitions of each specimen were performed to access the homogeneity of the prepared samples. For the analysis of the chemical functional groups of interest, a deconvolution was made in the original spectra through Origin^®^ software (version 6.1, OriginLab, Northampton, MA, USA). Due to the use of the same force all spectra were normalized and the area of specific bands used for the determination of the degree of conversion of the double bonds.

The thermal events of the specimens, especially T_g_, were determined by differential scanning calorimetry (DSC, TA Instruments, Newcastle, USA) in a DSC Q100 V9.9 equipment, with a heating rate of 10 °C.min^−1^ and with a constant nitrogen flow of 50 mL.min^−1^. The T_g_ values were determined through the maximal height of the 1st derivative of each obtained curve.

Three-point bending (3PB) static tests were performed with the spam of 40 mm, according to ASTM Standard D 7264/D 7264M-07 [23], and a Shimadzu AG-10 universal testing machine (Shimadzu Corp., Kyoto, Japan) equipped with a 10 kN load cell was used. For each condition, at least five specimens were tested at room temperature and a rate of 2 mm.min^−1^.

The flexural properties were also obtained according to ASTM Standard D 7264/D 7264M-07 [23] and, for example, while the flexural strength was calculated as the nominal stress at the middle span section, using the maximum value of the load Equation (1), the bending stiffness modulus was obtained by linear regression of the load–displacement curves considering the interval in the linear segment and according to Equation (2). A correlation factor more significant than 95% was used. Finally, the flexural strain (*ε*) was calculated according to Equation (3).

(1) $$\sigma =\frac{3\;P\;L}{2\;b\;h^{2}}$$

(2) $$E=\frac{\Delta P\cdot L^{3}}{48\Delta u\cdot I}$$

(3) $$\varepsilon_{f}=\frac{6\times S\times h}{L^{2}}$$

where *P* is the load, *S* the deflexion, *L* the span length, *b* the width, *h* the thickness of the specimen, *I* the moment of inertia of the cross-section and ∆*P* as well as ∆*u*, respectively, the load range and flexural displacement range in the middle span for an interval in the linear region of the load versus displacement plot.

The same equipment (Shimadzu AG-10) was used to carry out stress relaxation (SR) and creep tests at room temperature and with similar specimens to those shown in Figure 1. In the first case, all experimental procedure was supported by ASTM D6048-07 [24], where a fixed strain was applied (correspondent to around 51.5 MPa for all configurations) and the stress recorded during the loading time of 10,800 s. For this study, only specimens cured at room temperature (method 1) and with post-cure at 40 °C for 24 h (method 5) and 60 °C for 24 h (method 6) were analyzed. Finally, this bending strain value was selected to guarantee that all SR tests were carried out in the elastic regime of all conditions studied. The creep tests were carried out according to the procedures described in ASTM D7337M-12 [25], at room temperature, for constant stress of 20 MPa, and the displacement recorded during the loading time. In both cases, the data were analyzed according to the respective ASTM standards. On the other hand, and as reported in the open literature [26,27,28], these short-term tests reveal to be an easy, fast, and reliable method to predict long-term behavior. In fact, due to the main costs incurred with long-term test programs, in addition to the fact that long periods between the development of new or improved materials and their applications are not allowed, it is highly desirable that the design data could be obtained by extrapolating the results obtained by short-term tests.

## 3. Results and Discussion

**Figure 2:**
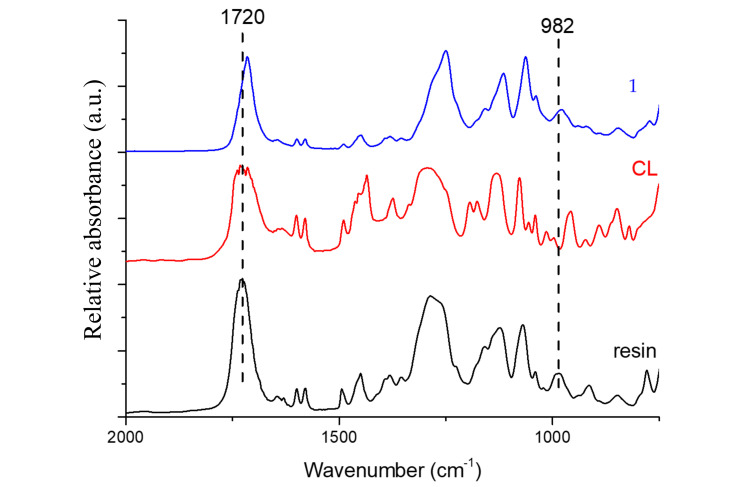
FTIR spectra of the resin, crosslinker (CL) and sample subjected to curing parameter 1.

An analysis of the chemical modifications was carried out, considering the different curing parameters. Figure 2 shows the FTIR spectra of the polyester resin, crosslinker agent (CL), and curing process at room temperature (method 1). Only the absorptions bands of the stretching mode of carbonyl groups (>C = O) at 1720 cm^−1^, and the polyester C = C bonds (982 cm^−1^), as assigned by other researchers [12,29,30,31], will be considered.

**Figure 3:**
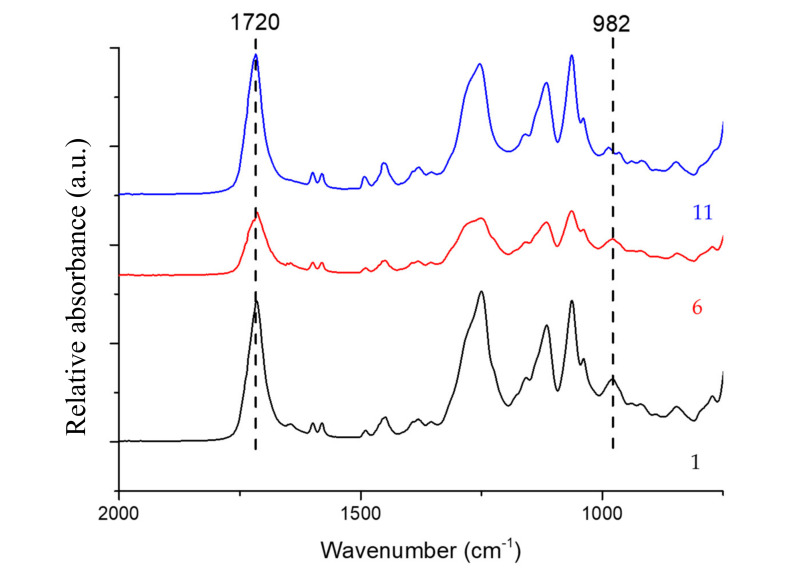
FTIR spectra of samples cured according to parameters 1, 6, and 11.

**Figure 4:**
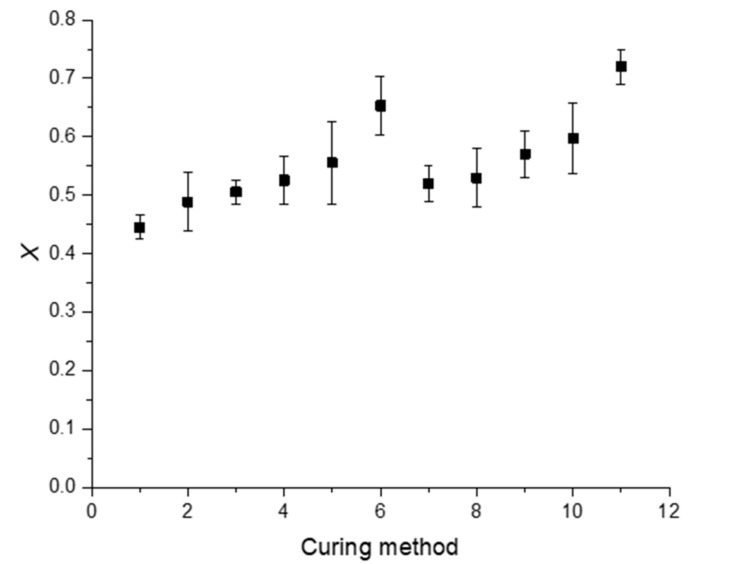
Degree of conversion of C = C polyester bonds (*X*) according to the curing parameters.

The normalized ratio of the absorption between the mentioned peaks, taking the peak of the carbonyl group as an internal reference, after curing (Figure 3), allows estimating the degree of conversion of the C = C from polyester bonds (*X*), according to previous studies [32]. The results are displayed in Figure 4 and can provide a rough approximation of the conversion of double bonds. However, it must be highlighted that the covalent bond between adjacent macromolecules can occur via other chemical reactions that do not involve the polyester unsaturated C = C bonds.

It is possible to conclude that a higher degree of conversion was obtained when a higher temperature was used at the beginning of the curing process, as suggested by the comparison between the values from methods 1, 6, and 11. The use of the first step of 24 h curing at room temperature followed by different curing times at higher temperatures (specimens 2–5 and 7–10), induced an almost linear increase of the degree of conversion. These results suggest that the RT induced an increase of the mixture viscosity that makes the evolution of further crosslinking in the post-curing stage difficult. The results suggest that to obtain a higher degree of conversion of the double bonds it is preferable to start with a higher temperature of the curing reaction, instead of a lower temperature followed by higher temperatures in the post-curing step.

**Figure 5:**
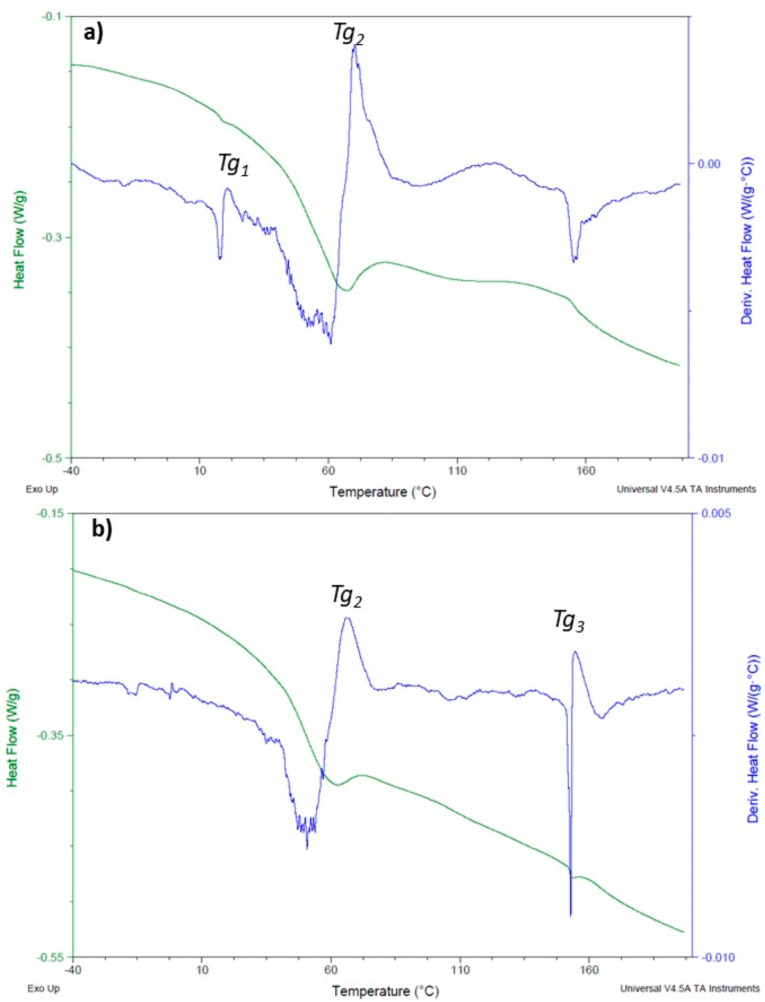
DSC heat flows and derivatives of specimens 1 (**a**) and 11 (**b**), showing the T_g_ values determination.

In the literature, at least for the last three decades, DSC studies are used to more accurately calculate the percentage of the crosslinking degree [33,34,35,36]. However, the reported studies usually refer to the system with a complete crosslinking reaction, which, as stated before, will not occur for the used resin nor for the tested temperatures. Nevertheless, in this work the DSC values provided additional information through the T_g_ determination (Table 2 and Figure 5).

The results indicate that when the curing process was made at room temperature, a T_g1_ around 16 °C always appeared. This indicates that the system presents free styrene molecules that are not crosslinked. They contribute to a high free volume of the system [33], which, consequently, presents a low (T_g1_). Even with a post-cure step at 40 °C (specimens 2–5) this endothermal transition occurred. For higher times of post-curing at 40 °C the system began to present a T_g_ value, around 144 °C (T_g3_), which is the main indicator of the crosslinking reaction. This first T_g_ disappeared when the curing process was made at 40 or 60 °C with or without subsequently post-curing (specimens 6–11) indicating the low crosslinking efficiency of the process when the curing step was made at room temperatures. These results reinforce the observations made after the FTIR characterization and, according to the literature the process is limited by the decrease of the diffusion of the reactive species, because radicals are trapped and the propagation reaction is stopped [36]. For the system that begins with the curing temperature of 60 °C the post-cure procedure has very little effect on the crosslinking percentage, as the T_g3_ increased for 0 h or 24 h of post-curing, at RT, was not significant.

**Figure 6:**
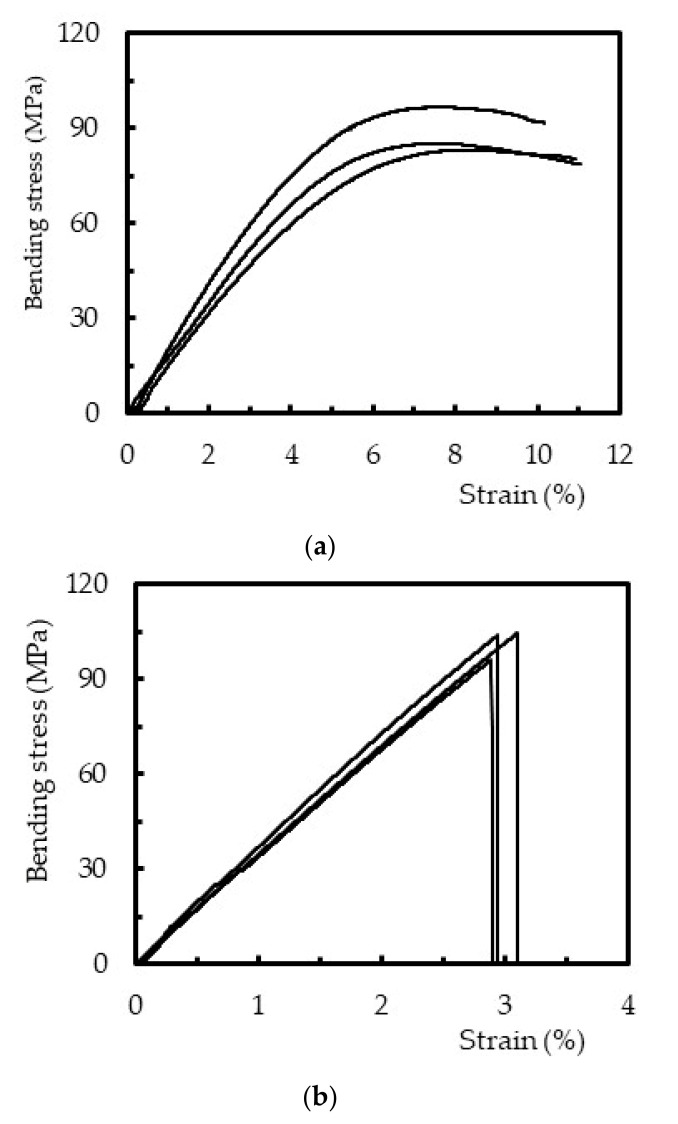
Flexural stress versus strain for: (**a**) post-cure at 40 °C for 12 h (specimen 4) and (**b**) post-cure at 60 °C for 6 h (specimen 8).

Flexural static tests were performed to obtain the bending properties against the different cure procedures (different exposure times and temperatures). In this context, Figure 6 presents typical bending stress versus strain curves obtained for each condition, which are representative of all other methods.

Even though Figure 6a represents the results obtained for curing parameters 4 (post-curing at 40 °C for 12 h), it is representative of all the specimens that involved the curing temperature of 40 °C. In this case, (Figure 6a) all curves present two distinct regions: a linear behavior up to around 4–5% of the bending strain, followed by a non-linear region in which the maximum bending stress occurs. These two regions appeared in all samples cured or post-cured at 40 °C, where the strain corresponding to the maximum bending stress occurred between 2% and 4%. However, the bending failure strain reached values above 10% due to the low crosslinking density [19,22]. According to Figure 4, the complete crosslinking has not yet been reached and, consequently, a certain level of molecular mobility is expected.

On the other hand, for specimens where the curing temperature of 60 °C was involved, all curves show a nearly fragile behavior. In these conditions, a linear region up to the maximum load was observed, followed by a significant drop, due to reduced molecular mobility promoted by the high density of crosslinking [19,22]. As reported in Figure 4 and Table 2, and comparatively to the value obtained at room temperature, the degree of conversion was around 1.4 times higher for 40 °C and 1.6 times higher for 60 °C. Therefore, a higher degree of conversion was obtained for higher temperatures and, in this case, the bending failure strain coincided with the maximum bending stress, typical behavior of a brittle material.

**Figure 7:**
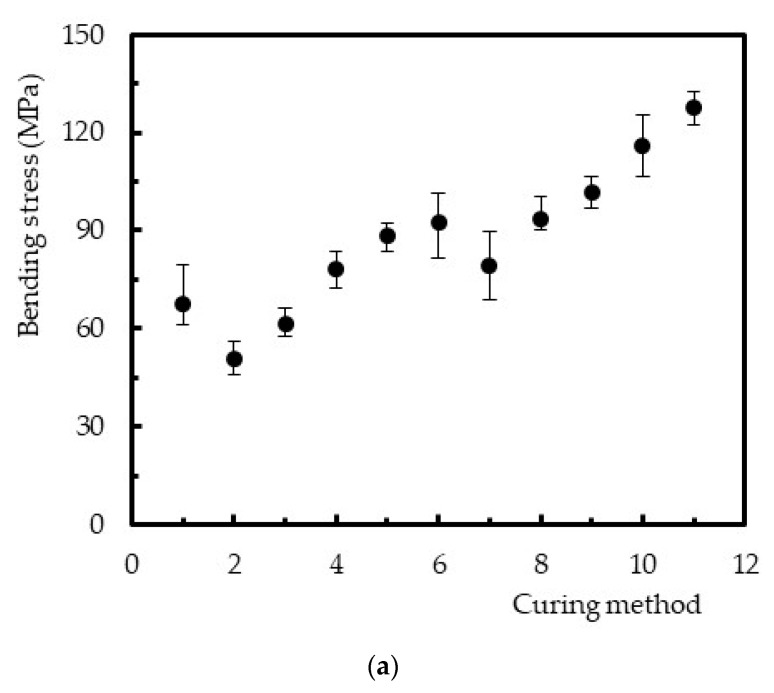

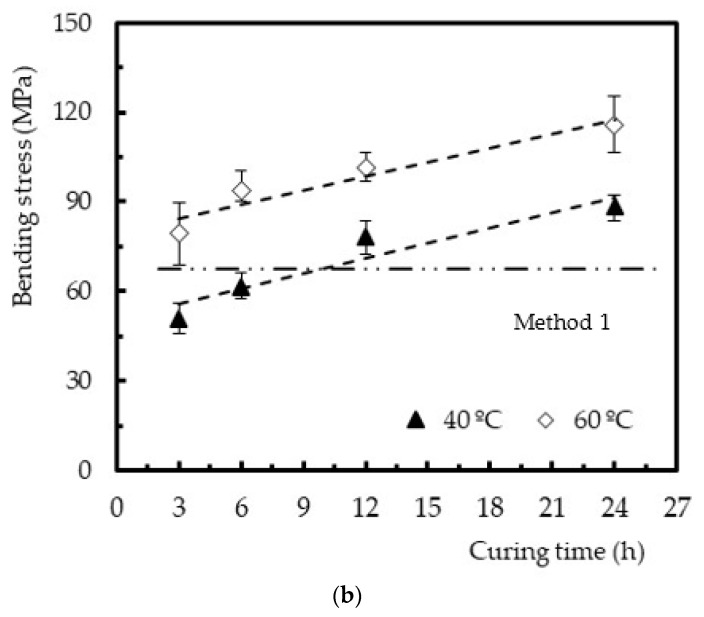
(**a**) Effect of the curing parameters on the bending stress and (**b**) effect of the post-cure on the bending stress.

Figure 7 presents the maximum bending stress against the curing parameters in terms of average values (marks), and the dispersion bands indicate the maximum and minimum values for each condition. It is possible to observe that the curing parameters had a significant influence on the maximum flexural strength of the polyester resin.

From Figure 7a, for example, it is noticed that the use of higher temperatures in the curing process, and without post-cure (specimens 6 and 11), was responsible for higher bending strengths. Compared to the resin cured at room temperature (specimen 1), the bending strength increased around 36.5% for 40 °C (specimen 6) and 88.6% for 60 °C (specimen 11) due to the higher degree of conversion of unsaturated C = C bonds. Therefore, the lower molecular mobility, promoted by the higher conversion of double bonds, is responsible for the higher bending strengths [19,22].

On the other hand, in the case of post-cures effect, the viscosity induced by the crosslinking at RT makes further reticulation when the temperature is increased during the second stage of the process difficult. This behavior is explained by the diffusion problems of the crosslinking agent within the reticulated structure. Regardless of the curing temperatures, Figure 7b confirms what was observed in Figure 4, and a linear increase in bending strength was observed as a function of the exposure time. A higher degree of conversion was achieved for higher exposure times, which, in principle, implies lower molecular mobility [19,22]. For example, the bending strength obtained for a post-cure at 40 °C for 24 h was 73.9% higher than the value obtained at 40 °C for 3 h. At 60 °C post-cure temperature, the increase was around 46.1%.

**Figure 8:**
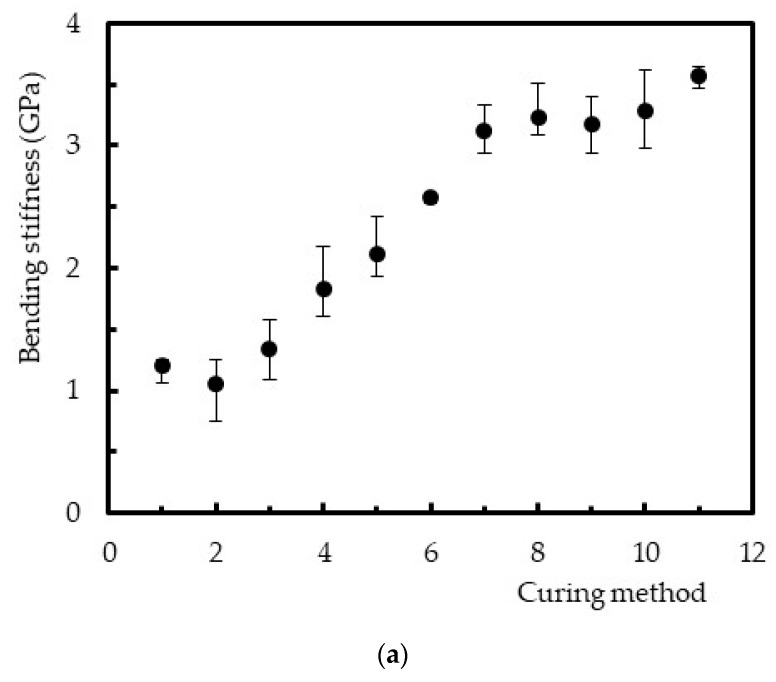

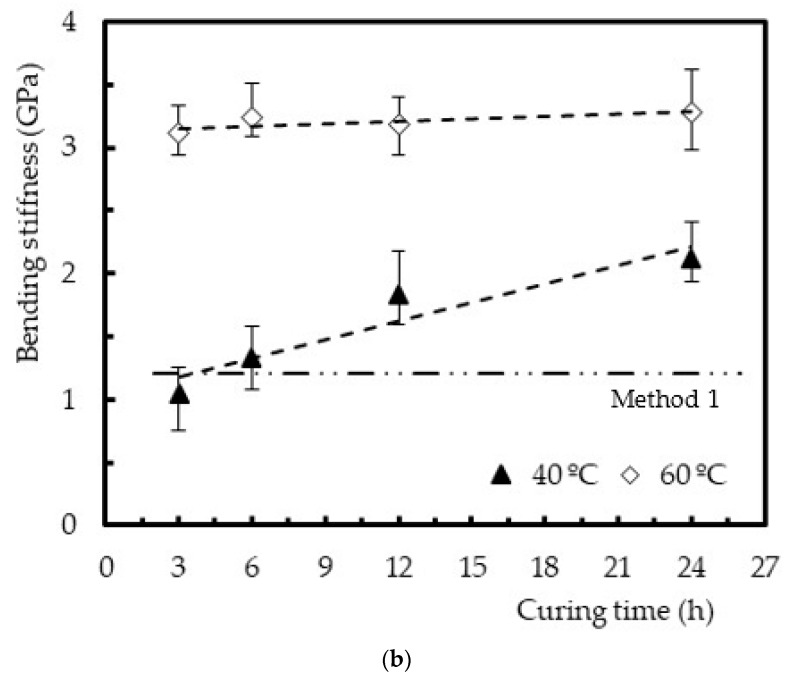
(**a**) Effect of the curing parameters on bending stiffness and (**b**) effect of the post-cure on bending stiffness.

Figure 8 shows the post-cure effect on bending stiffness, and a behavior very similar to that obtained for bending strength was observed.

In more detail, from Figure 8a, it is possible to observe that, for methods without post-cures (methods 1, 6, and 11), an increase in temperature was responsible for higher bending stiffness values. Bending stiffness was about two times higher for 40 °C (method 6) and three times higher for 60 °C (method 11), compared to the value obtained at room temperature. As reported above, and according to Figure 4 and Table 2, the lower molecular mobility due to a higher density of crosslinking explains these improvements [19,22]. However, when the post-cure effect was analyzed separately in Figure 8b, one more time, a linear increase was observed as a function of the exposure time. Higher exposure times promote a higher degree of conversion of the unsaturated ester bonds, and, consequently, less molecular mobility [19,22]. The bending stiffness obtained for a post-cure at 40 °C for 24 h was 73.3% higher than the value obtained at 40 °C for 3 h, and for the same comparison at 60 °C this value was only 5.5% higher. It is noticed that the exposure time was less expressive in this property with increasing temperature. A brief comparison with the study developed by Gudapati et al. [37], carried out with a cure of 24 h at RT followed by 2 h at 80 °C, evidences a bending modulus similar to the value obtained with method 3 (24 h at RT followed by a post-cure performed at 40 °C for 6 h). In this context, it is noticed the post-cure temperature effect on the crosslinking and, consequently, on the mechanical properties. As reported above, it is the most important factor that influences the extent of cross linking [8]. However, in terms of flexural strength, these authors obtained lower values.

**Figure 9:**
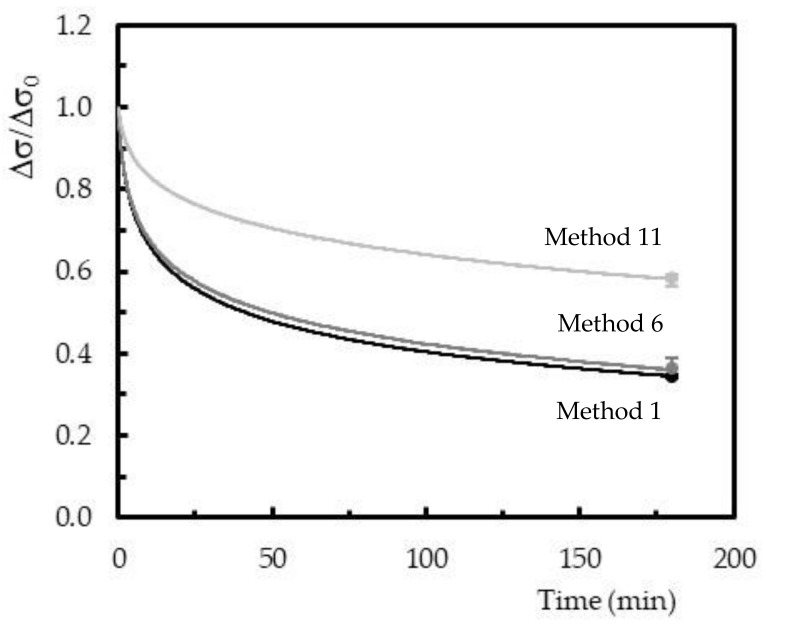
Stress relaxation curves obtained for the specimens 1, 6, and 11 (bending strain correspondent to 51.5 MPa).

Considering the viscoelastic behavior, Figure 9 presents the stress relaxation curves only for the methods without the post-cure. This figure plots the average bending stress versus time, where σ is the bending stress at any given moment of the test, and σ_0_ is the initial bending stress. The final bands represent the maximum and minimum values obtained for each condition analyzed.

In general, the stress decreased over time to one value expected to be constant, but in the present work, this constant bending stress was not achieved because it focuses on short-term tests. These tests represent an easy, fast, and reliable method to predict long-term behavior [26,27,28]. Another similarity with the open literature is the existence of an initial stage, in which the stress decreases considerably with the remaining time [38,39,40]. The duration of this initial stage depends significantly on the material. In the present case, for example, considering the values obtained for method 11, stress decreased around 29.5% after 50 minutes, but this value reached 17.7% in the last 130 minutes. Regarding method 6, these values were 50.2% and 27.5%, while for method 1 they were 52.3% and 27.9%, respectively. This result was clear evidence that the bending stress was tending to a constant value, as reported above.

The curing process’ effect was also analyzed at the level of difference observed between the initial bending stress value and the final one after 3 hours. It was possible to conclude that, for curing at room temperature, the bending stress decreased around 65.6% concerning the initial value. However, this value was 63.9% for 40 °C and 42% for 60 °C. This decrease is well reported in the open literature, which is based on physical phenomena (molecular rearrangements that require little formation or rupture of primary bonds) and/or chemical phenomena (chain scission, crosslink scission, or crosslink formation) [41,42,43]. Both were expected in this study, but it is possible to conclude that higher curing temperatures decrease stress relaxation behavior. As reported in the static properties, also the best performance in terms of stress relaxation was obtained for a temperature of 60 °C. The degree of conversion, as shown in Figure 4, was 1.2 times higher than the one observed at 40 °C, and, consequently, this resin system had less molecular mobility.

**Figure 10:**
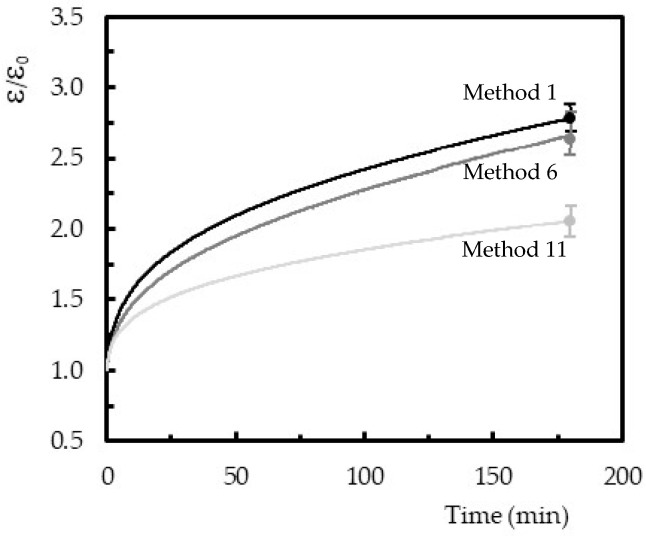
Creep curves obtained for the methods 1, 6, and 11 (bending stress of 20 MPa).

The reasons that explain the stress relaxation behavior are the same that support the creep curves shown in Figure 10, where ε represents the strain value obtained at any instant of the test, and ε_0_ is the initial instantaneous strain (value obtained immediately after application of the respective load). The final bands represent the maximum and minimum values obtained for each condition analyzed. It is possible to observe that all curves present an instantaneous strain, followed by the primary and secondary creep regimes that characterize the typical creep curves. In this study, the third regime was expected to occur only for higher stress values or longer times. Finally, the strain increased with time; however, this behavior became less pronounced with the increasing of curing temperature. For example, while for control samples (method 1), the initial strain was around 3.2 times higher concerning its initial value, this value decreased to 2.9 times for 40 °C and to 2 times for 60 °C. In this case, the curing temperature of 60 °C promoted a decrease of the creep strain, after 180 mm, around 37.5% concerning the resin cured at room temperature. 

The creep phenomenon in polymers occurs even at room temperature and for stresses below their ultimate strength due to the molecular motion in the backbone polymer arrangement [44,45,46,47]. Therefore, the degree of conversion 1.4 times higher for 40 °C and 1.6 times for 60 °C, when compared to the value obtained at room temperature, explains the higher crosslinking rate and, consequently, the lower molecular mobility in the resin. In such a context, lower creep strain values are expected/obtained.

Finally, the ability to model the viscoelastic behavior of polymer-based materials is determined to predict their long-term structural behavior. In terms of stress relaxation, for example, literature reports complex models to the detriment of those based on spring-dashpot systems, because the data are not fitted by a linear function. Therefore, the Kohlrausch–Williams–Watts (KWW) function, an empirical “stretched exponential” function, is suggested by the open literature as an appropriate methodology to model the stress relaxation [27,28,39,40,48,49]. According with this model, the relaxation function ∅ as a function of time is given by Equation (4):

(4) $$\emptyset =\;\frac{\sigma \left(t\right)}{\sigma_{0}}=e^{-{\left(\frac{t}{\tau }\right)}^{\beta }}$$

where *σ*(*t*) and *σ*_0_ are the stress at time *t* and at *t* = 0, respectively, *β* a fractional power exponent (known as non-exponential factor), and *τ* the KWW relaxation time.

**Figure 11:**
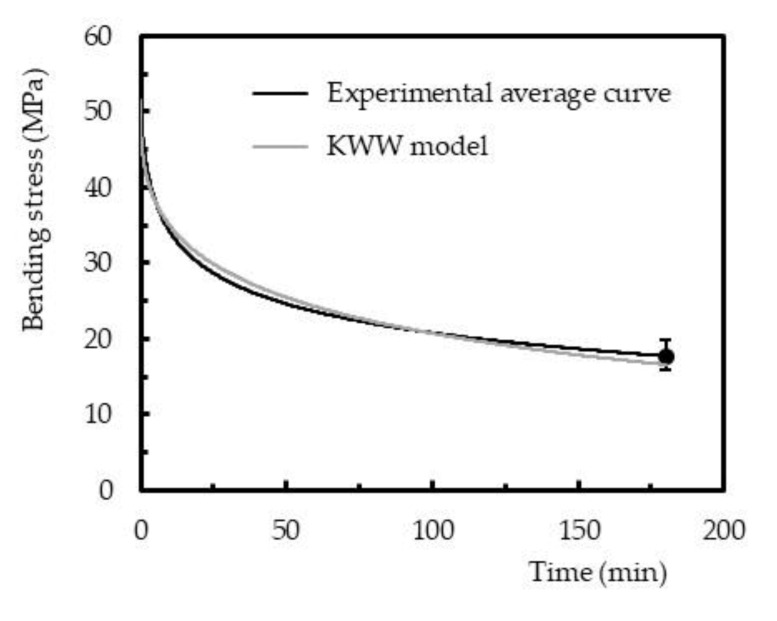
Comparison between the experimental average curve and theoretical curve obtained with the Kohlrausch–Williams–Watts (KWW) model for samples cured by method 1 (bending strain correspondent to 51.5 MPa).

Figure 11 compares the experimental results against the theoretical ones obtained with the KWW model for samples cured by Method 1. These curves are representative of all conditions and, apparently, the Kohlrausch–Williams–Watts (KWW) function fits the data successfully. Table 3 reports all parameters of the KWW model and its accuracy, where the errors observed between the average experimental curve and the KWW prediction were less than 8.9% (in absolute value) after 3 h of testing. Therefore, it is possible to conclude that the model fits to the data successfully.

Regarding the creep behavior, the Findley power law model is one of the most widely used to describe this phenomenon in composite materials and has been recommended, inclusively, by the ASCE Structural Plastics Design Manual to analyze and design the long-term strength [50]. The Findley’s power law is given by Equation (5):

(5) $$\varepsilon \left(t\right)=\varepsilon_{0\;}+\;At^{n}$$

where *ε*(*t*) is the creep displacement at time *t*, *ε*_0_ is the instantaneous elastic displacement or time-independent, *A* is the amplitude of transient creep (time-dependent), and *n* is a constant independent of the stress and generally less than one [51]. Several studies can be found in the open literature, where the Findley’s power law was also applied to short-term creep data [27,28,51,52,53].

**Figure 12:**
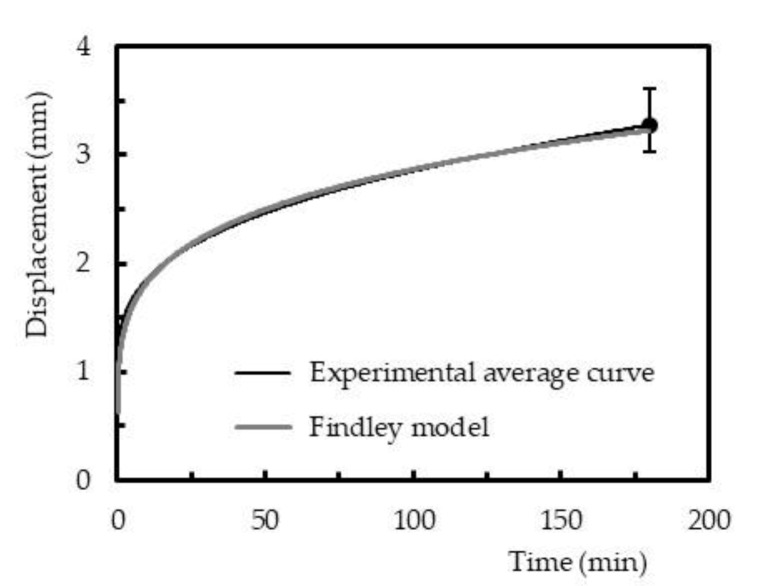
Comparison between experimental average curve and theoretical curve obtained with the Findley power law model for samples cured by method 1 (bending stress of 20 MPa).

Therefore, similar to the previous figure, Figure 12 compares the experimental results against the theoretical ones obtained with the Findley power law model for samples cured by method 1. These curves are representative of all the conditions studied, and it is noticed that the Findley model fits the data successfully. Table 4 reports all parameters of the Findley power law model and its accuracy after 3 h of testing. The maximum error observed between the average experimental curves and the predicted ones was less than 7.1%, for all conditions studied, which confirms that the model fits the experimental data successfully.

## 4. Conclusions

The main goal of this study was to analyze the effect of the curing parameters on the mechanical properties of a polyester resin usually used in surfboards. For this purpose, the curing temperature was lower than the normally used with this type of resins, for other applications, in order to obtain lower percentages of curing and evaluate their influence on the mechanical properties. Moreover, the use of lower temperatures also has a beneficial impact both to the economic and environmental aspects. Three-point bending tests were performed to assess the bending properties and both stress relaxation and creep behavior.

The polymer if cured at room temperature, even when followed by short times of post-curing at 40 °C, presents unreacted macromolecules according to the DSC characterization. The higher percentages of crosslinking were obtained when the curing process was made at 60 °C for 24 h.

In terms of static properties, it was also possible to conclude that using higher curing temperatures, without post-curing, is preferable to the approach that involves curing followed by post-curing, because the exposure time at room temperature induces a percentage of conversion of double bonds that difficult further crosslinking in the second process step (post-cure). Therefore, the maximum bending strength and modulus were obtained at 60 °C for 24 h. Finally, it was also noticed that, when a post-cure was applied, these properties increased linearly with the exposure time due to a higher degree of conversion achieved.

Finally, the viscoelastic behavior was evaluated, and both stress relaxation and creep strain presented the lowest values for samples cured at 60 °C. The degree of conversion was higher for 60 °C, and, consequently, this resin system had less molecular mobility due to its higher crosslinking. The Kohlrausch–Williams–Watts (KWW) equation and the Findley power law model were used to predict the viscoelastic behavior, and good accuracy was obtained between the experimental results and those predicted by the proposed models.

## Figures and Tables

**Table 1:** Summary of cure and post-cure conditions.

Cure Method	Cure		Post-Cure	
	Time (h)	Temperature (°C)	Time (h)	Temperature (°C)
1	360	RT	-	-
2	24	RT	3	40
3	24	RT	6	40
4	24	RT	12	40
5	24	RT	24	40
6	24	40	-	-
7	24	RT	3	60
8	24	RT	6	60
9	24	RT	12	60
10	24	RT	24	60
11	24	60	-	-

RT = Room temperature.

**Table 2:** Average and standard deviation values of T_g_ calculated from differential scanning calorimetry (DSC).

Curing Parameters	T_g_ Values (°C)		
	T_g1_	T_g2_	T_g3_
1	19.5 ± 0.8	66.8 ± 0.2	n.d.
2	18.7 ± 1.5	66.4 ± 0.3	n.d.
3	18.0 ± 0.7	66.6 ± 0.6	n.d.
4	17.7 ± 1.0	55.8 ± 0.2	n.d.
5	17.8 ± 1.0	63.5 ± 0.3	143.7 ± 2.0
6	n.d.	62.6 ± 0.3	159.1 ± 0.1
7	n.d.	62.1 ± 0.5	162.0 ± 1.0
8	n.d.	58.3 ± 0.9	162.8 ± 0.7
9	n.d.	62.3 ± 0.3	163.3 ± 0.3
10	n.d.	62.5 ± 1.0	165.2 ± 0.8
11	n.d.	62.1 ± 0.0	157.4 ± 0.2

n.d. = not detected.

**Table 3:** Parameters of the KWW model for stress relaxation.

Curing Method	*β*	*τ*	Bending Stress after 3 h (MPa)		
			Experimental Value	KWW Value	Error (%)
1	0.372	1.29 × 10^2^	17.7	16. 6	6.2
6	0.393	2.83 × 10^2^	19.4	21.3	8.9
11	0.409	7.12 × 10^2^	29.9	29.1	2.7

**Table 4:** Parameters of the Findley power law model for creep.

Curing Method	*ε*_0_	*A*	*n*	Displacement after 3 h (MPa)		
				Experimental Value	Findley Value	Error (%)
1	0.229	0.397	0.218	3.3	3.2	3.0
6	0.231	0.378	0.199	2.5	2.6	3.8
11	0.177	0.250	0.166	1.4	1.3	7.1
